# Causes of altered ventricular mechanics in hypertrophic cardiomyopathy: an in-silico study

**DOI:** 10.1186/s12938-021-00900-9

**Published:** 2021-07-22

**Authors:** Ekaterina Kovacheva, Tobias Gerach, Steffen Schuler, Marco Ochs, Olaf Dössel, Axel Loewe

**Affiliations:** 1 Institute of Biomedical Engineering, Karlsruhe Institute of Technology (KIT), Kaiserstr. 12, 76131 Karlsruhe, Germany; 2 Department of Cardiology, Theresienkrankenhaus, Academic Teaching Hospital of Heidelberg University, Bassermannstr.1, 68165 Mannheim, Germany

**Keywords:** Hypertrophic cardiomyopathy, In-silico study, Altered mechanics, Active and passive forces, Wall thickness, Fiber disarray, Strain, Strain rate

## Abstract

**Background:**

Hypertrophic cardiomyopathy (HCM) is typically caused by mutations in sarcomeric genes leading to cardiomyocyte disarray, replacement fibrosis, impaired contractility, and elevated filling pressures. These varying tissue properties are associated with certain strain patterns that may allow to establish a diagnosis by means of non-invasive imaging without the necessity of harmful myocardial biopsies or contrast agent application. With a numerical study, we aim to answer: how the variability in each of these mechanisms contributes to altered mechanics of the left ventricle (LV) and if the deformation obtained in in-silico experiments is comparable to values reported from clinical measurements.

**Methods:**

We conducted an in-silico sensitivity study on physiological and pathological mechanisms potentially underlying the clinical HCM phenotype. The deformation of the four-chamber heart models was simulated using a finite-element mechanical solver with a sliding boundary condition to mimic the tissue surrounding the heart. Furthermore, a closed-loop circulatory model delivered the pressure values acting on the endocardium. Deformation measures and mechanical behavior of the heart models were evaluated globally and regionally.

**Results:**

Hypertrophy of the LV affected the course of strain, strain rate, and wall thickening—the root-mean-squared difference of the wall thickening between control (mean thickness 10 mm) and hypertrophic geometries (17 mm) was >10%. A reduction of active force development by 40% led to less overall deformation: maximal radial strain reduced from 26 to 21%. A fivefold increase in tissue stiffness caused a more homogeneous distribution of the strain values among 17 heart segments. Fiber disarray led to minor changes in the circumferential and radial strain. A combination of pathological mechanisms led to reduced and slower deformation of the LV and halved the longitudinal shortening of the LA.

**Conclusions:**

This study uses a computer model to determine the changes in LV deformation caused by pathological mechanisms that are presumed to underlay HCM. This knowledge can complement imaging-derived information to obtain a more accurate diagnosis of HCM.

**Supplementary Information:**

The online version contains supplementary material available at 10.1186/s12938-021-00900-9.

## Background

Hypertrophic cardiomyopathy (HCM) is a relatively common inherited disorder, with a prevalence of 1:500, which develops in the absence of an identifiable cause [1, 2]. There are several phenotypes of HCM, depending on the localization and distribution of hypertrophy in the heart: asymmetric, symmetric/concentric, apical, or mid-ventricular obstruction [3]. HCM results in an increased ratio of wall to lumen volume, which can be diagnosed by echocardiographic or magnetic resonance assessment of left-ventricular anatomy [1]. Besides this morphological modification, further abnormalities are underlying the HCM phenotype.

In HCM hearts, fibrosis and myocardial cell disarray can be present and might have evolved for years before the onset of symptoms [1]. The disarray of the cells can be quantified by fractional anisotropy (FA)—a measure obtained by diffusion tensor magnetic resonance imaging (DT-MRI) or shear wave imaging (SWI). Ariga et al. [4] reported reduced FA in HCM patients compared to control subjects, measured by DT-MRI. Villemain et al. [5] reported similar findings in pediatric HCM patients using SWI.

Further structural abnormalities in HCM were detected by SWI on an organ level: passive ventricular stiffness was significantly higher in HCM compared to the control group [5]. In HCM, increased stiffness on the organ level could not be explained by an alteration in the viscoelastic properties of the cardiac myocytes, since the passive stiffness of prepared HCM cardiac myocytes was measured to be the same as healthy donor myocytes [6]. The stiffer tissue behavior might be due to further factors such as cell disarray or tissue fibrosis. Furthermore, the maximal active force was markedly lower in HCM myocytes than in donor myocytes [6]. In clinical routine, it is not possible to measure active force development of the myocytes in-vivo. Furthermore, the application of SWI for stiffness measurement is limited to the entire ventricle and might be not applicable for all patients [7]. A reconstruction of the myocardial cell orientation with DT-MRI is very time-consuming and delivers limited anatomical coverage of the ventricle [8].

These limitations of the available imaging modalities make it impossible or at least cumbersome to identify abnormalities underlying the HCM phenotype in clinical routine. Nevertheless, the consequences of these structural changes can be measured and quantified to provide a basis to diagnose HCM. This diagnosis is often based on echocardiographic assessment of the systolic and diastolic function of the left ventricle (LV) [1] and parameters derived from tissue imaging (strain and strain rate) [9]. Furthermore, MRI can quantify heart motion and function by cine imaging, which enables LV wall thickness calculation. Tissue phase mapping and feature tracking provide LV radial, circumferential, and longitudinal myocardial velocity time courses, as well as global and segmental systolic and diastolic peak velocities [10]. The longitudinal strain in the left atrium (LA) is measured as well to inform HCM diagnosis [11]. Such precise assessment of the cardiac function enables the quantification of altered mechanics in HCM patients compared to healthy volunteers.

Concurrently to these advancements in imaging modalities in the past decades, the field of computational cardiac modeling has progressed to provide an accurate and robust in-silico representation of the human heart beat  [12–16]. In a numerical study, Usyk et al. [17] investigated the influence of different structural properties of disarrayed myocardium using a three-dimensional finite-element model of systolic contraction. They created an ellipsoid model of the normal and the hypertrophied ventricle and altered the passive material parameters to examine their sensitivity on the systolic strains. In our work, we used a four-chamber model to represent the motion of the whole heart. Ubbink et al. [18] employed a finite-element model of cardiac mechanics to investigate the influence of the myofiber orientation on the circumferential and circumferential-radial shear strain. Further numerical studies were performed to quantify the impact of structural changes of the myocardium on the model prediction. Campos et al. [19] performed a sensitivity analysis considering uncertainties in wall thickness, in the material properties, and fiber orientation based on a 17-American Heart Association (AHA) segments diagram. In [20], Campos et al. additionally incorporated uncertainties in active stress and the circulatory model to quantify their impact on the stress, strain, and global deformation parameters of the LV. The variations in the fiber orientation were achieved by changing the angles used as an input for the fiber generation algorithms. In contrast, we included fiber disarray in the mid-wall as measured in HCM hearts. The effect of uncertainties in material input parameters on cavity volume, the elongation and radius of the ventricle, wall thickness, and the rotation was studied by Osnes et al. [21] on an ellipsoid geometry. Such in silico studies can help to understand the relationships between the structural changes of the tissue and the ventricular mechanics.

In our study, we are particularly interested in modeling HCM hearts and establishing cause–effect relationships between previously described pathological mechanisms in HCM hearts and their effect on ventricular mechanics. The identification of distinct underlying abnormalities leading to the altered mechanical behavior of HCM hearts complementary to imaging could be valuable information for clinicians on the way to clearer and faster diagnoses. It can provide directions to differentiate HCM from other cardiac conditions, in which thickened walls are present. Moreover, it could help to separate healthy hearts from HCM genotype-positive but phenotype-negative hearts.

In this work, we conduct in-silico experiments to identify potential underlying causes of altered ventricular mechanics observed in HCM patients. The numerical heart simulator includes models of active force development, passive stiffness, the circulatory system, and appropriate boundary conditions to conduct a sensitivity study. We alter model parameters capturing different pathological mechanisms in a virtual heart: increased wall thickness (WT) of the LV to represent concentric hypertrophy, increased tissue stiffness by a factor of 5, decreased active force development by 40%, and disarray of the fiber orientation (FO) in the LV mid-wall with reduced FA. We explore different combinations of these mechanisms to analyze their effect on ventricular mechanics (Table 1). Furthermore, we compared a healthy control heart simulation and a simulation comprising all potential HCM mechanisms in terms of several evaluation metrics defined in detail in "Methods" section.

## Results

In the following, we describe the observed alteration of the mechanical behavior of the in-silico heart due to variations in the input parameters of the computational model. Table 1 provides an overview of the cases covered in the sensitivity analysis. Each case is defined by a combination of the four model variants described in detail in "Methods" section.

**Table 1:** Overview of the cases of the sensitivity analysis and the corresponding variations of model components

Case	Geom. (mean WT ± std)	Passive force (*C*)	Active force ($T^{\text {V}}_{\text {max}}$/$T^{\text {A}}_{\text {max}}$)	FO
Case 1	Control ($10\,\pm \,2.3$ mm)	Control (309 Pa)	Control (100/35 kPa)	Control
Case 2	Control ($10\,\pm \,2.3$ mm)	Stiffened (1545 Pa)	Control (100/35 kPa)	Control
Case 3	Control ($10\,\pm \,2.3$ mm)	Control (309 Pa)	Decreased (60/21 kPa)	Control
Case 4	Control ($10\,\pm \,2.3$ mm)	Stiffened (1545 Pa)	Decreased (60/21 kPa)	Control
Case 5	HCM 1 (15 ± 3.3 mm)	Control (309 Pa)	Control (100/35 kPa)	Control
Case 6	HCM 2 (17 ± 4.1 mm)	Control (309 Pa)	Control (100/35 kPa)	Control
Case 7	HCM 2 (17 ± 4.1 mm)	Stiffened (1545 Pa)	Control (100/35 kPa)	Control
Case 8	HCM 2 (17 ± 4.1 mm)	Control (309 Pa)	Decreased (60/21 kPa)	Control
Case 9	HCM 2 (17 ± 4.1 mm)	Stiffened (1545 Pa	Decreased (60/21 kPa)	Control
Case 10	HCM 2 (17 ± 4.1 mm)	Control (309 Pa)	Control (100/35 kPa)	Disarray
Case 11	HCM 2 (17 ± 4.1 mm)	Stiffened (1545 Pa)	Control (100/35 kPa)	Disarray
Case 12	HCM 2 (17 ± 4.1 mm)	Control (309 Pa)	Decreased (60/21 kPa)	Disarray
Case 13	HCM 2 (17 ± 4.1 mm)	Stiffened (1545 Pa)	Decreased (60/21 kPa)	Disarray

Geom = geometry, *C* is the global stiffness in the myocardial tissue, $T^{\text {V}}_{\text {max}}$ is maximal active force in both ventricles, $T^{\text {A}}_{\text {max}}$ is maximal active force in both atria, and FO = fiber orientation

We evaluated wall thickening of the LV, the strain, strain rate, and velocity in radial, longitudinal, and circumferential directions of the LV. We calculated these measures regionally (in each of the 17 AHA segments) and globally (one value for the entire LV). Furthermore, we provide the LA longitudinal strain. For each global measure, we calculated the root-mean-squared deviation (RMSD). The RMSD for all cases and all metrics are provided in Additional file 1: Figures S1 and S2.

### Altered mechanics due to the wall thickness of LV

We quantified isolated changes of the LV WT by comparing the deformation for different geometries—control geometry (Case 1, WT $10\,\pm \,2.3$ mm), HCM 1 (Case 5, WT $15\,\pm \,3.3$ mm), and HCM 2 (Case 6, WT $17\,\pm \,4.1$ mm).

**Fig. 1:**
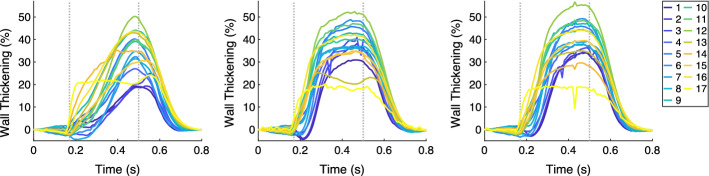
The time courses of the regional wall thickening (in each of the 17 AHA segments) for Case 1, 5, and 6 (left, middle, and right, respectively). In each plot, the first vertical line (at 0.17 s) indicates ED and the second line (at 0.5 s) ES. Case 1: control case (initial WT = 10 mm); Case 5: hypertrophic geometry (15 mm); Case 6: hypertrophic geometry (17 mm)

**Fig. 2:**
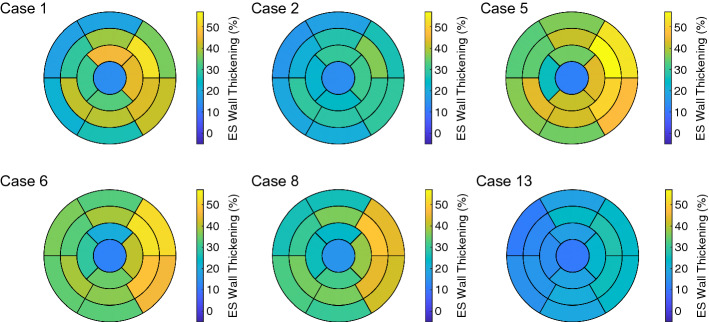
Bull’s-eye displays for Case 1, 2, 5, 6, 8, and 13 showing the wall thickening at ES. Case 1: control case; Case 2: increased stiffness; Case 5: hypertrophic geometry (15 mm); Case 6: hypertrophic geometry (17 mm); Case 8: hypertrophic geometry (17 mm), decreased active force; Case 13: virtual HCM case (all pathological changes included)

The increase of the regional WT for the hypertrophic geometries (Case 5 and Case 6) during systole, for all 17 segments, was faster compared to the one of the control geometry (Case 1). Between Case 5 and Case 6, no marked difference was observed (Fig. 1). Wall thickening at end-systole (ES) was in the same range in all three cases (Case 1: between 18.1 and 50.0%, Case 5: between 18.4 and 48.1%, and Case 6: between 18.1 and 51.3%). Nevertheless, the distribution among the segments changed—the thickening of the basal segments (1–6) increased as the initial WT increased. Figure 2 shows the ES distribution of the wall thickening.

**Fig. 3:**
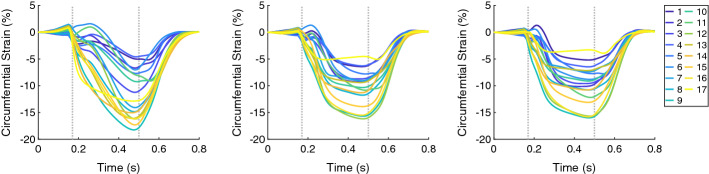
The time courses of the regional circumferential strain (in each of the 17 AHA segments) for Case 1, 5, and 6 (left, middle, and right, respectively). In each plot, the first vertical line (at 0.17 s) indicates ED and the second line (at 0.5 s) ES. Case 1: control case (initial WT = 10 mm); Case 5: hypertrophic geometry (15 mm); Case 6: hypertrophic geometry (17 mm)

**Fig. 4:**
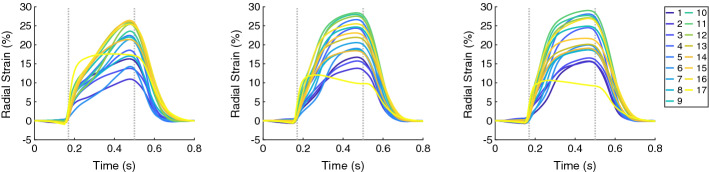
The time courses of the regional radial strain (in each of the 17 AHA segments) for Case 1, 5, and 6 (left, middle, and right, respectively). In each plot, the first vertical line (at 0.17 s) indicates ED and the second line (at 0.5 s) ES. Case 1: control case (initial WT = 10 mm); Case 5: hypertrophic geometry (15 mm); Case 6: hypertrophic geometry (17 mm)

The circumferential and radial strain and the strain rate (both global and segmental) for the control geometry differed from those of the hypertrophic geometries. An increase of radial strains and a decrease of circumferential strains for Case 1 were observed during the entire systole, while those for the hypertrophic geometries occurred during the first half of the systolic period (Figs. 3 and 4).

**Fig. 5:**
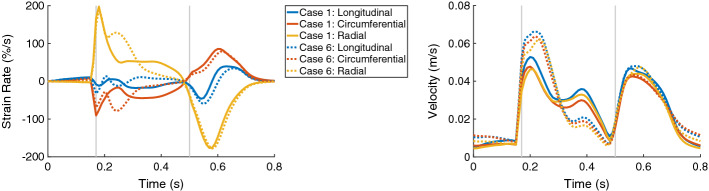
The time courses of the global strain rates (longitudinal, circumferential, and radial) are on the left and the global velocities (longitudinal, circumferential, and radial) are on the right, for Case 1 (solid lines) and Case 6 (dotted lines). In each plot, the first vertical line (at 0.17 s) indicates ED and the second line (at 0.5 s) ES. Case 1: control case (initial WT = 10 mm); Case 6: hypertrophic geometry (17 mm)

This can be derived also from the strain rates—they are higher for the radial direction and lower for the circumferential direction in the mid systole for the hypertrophic geometries compared to those in the control case. An initially similar strain rate is available for all three cases during the systole, with amplitude 200%/s for the radial strain rate and around 90%/s for the circumferential strain rate (Fig. 5, left). The regional strains at end-diastole (ED) in all three directions were comparable in all three cases.

**Fig. 6:**
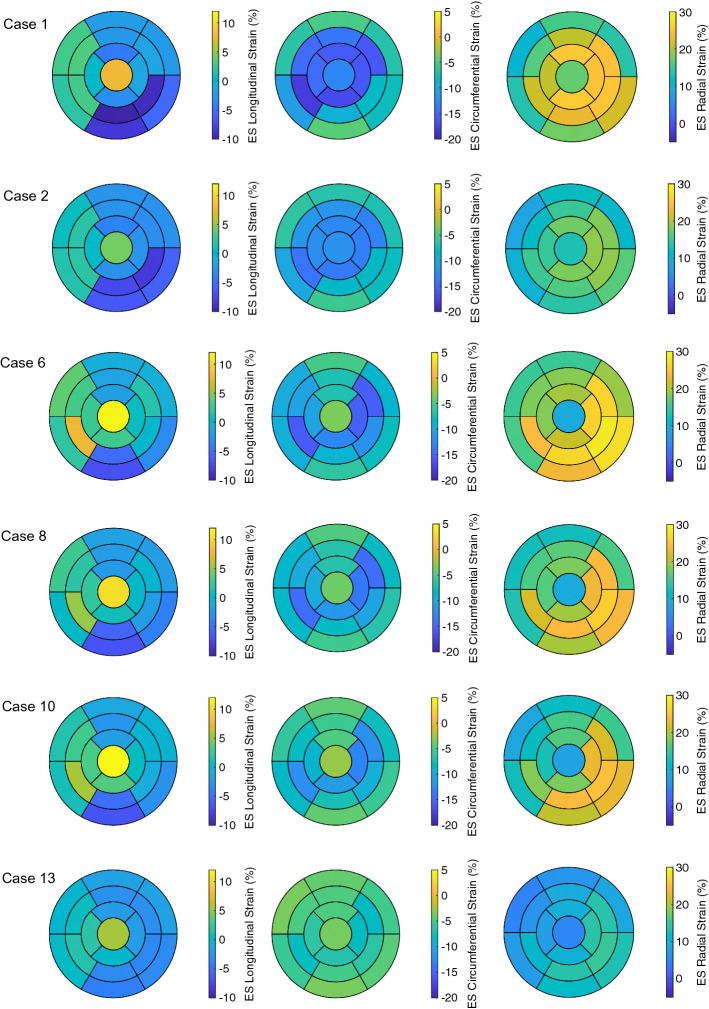
Bull’s-eye displays for Case 1, 2, 6, 8, 10, and 13 showing the longitudinal, circumferential, and radial strain at ES (first, second, and third columns, respectively). Each row corresponds to one case. Case 1: control case; Case 2: increased stiffness; Case 6: hypertrophic geometry (17 mm); Case 8: hypertrophic geometry (17 mm), decreased active force; Case 10: hypertrophic geometry, fiber disarray; Case 13: virtual HCM case (all pathological changes included)

The ES longitudinal strain indicated less shortening of the tissue since its minimum over the segments became higher when WT increased from −11.0% for Case 1 to around −6.5% for Case 5 and Case 6. Furthermore, the circumferential and radial strain at ES showed only minor differences when WT increased (Figs. 3 and 4). In Fig. 6, the distributions of the strains at ES are visualized for each local direction (longitudinal, circumferential, and radial).

The velocities in all local directions for the control case were differing from those of the hypertrophic geometries during the entire systole. The velocities for the control case were between 0.04 m/s and 0.05 m/s during the beginning and the middle of the systole, while the velocities for the hypertrophic geometries were high at the beginning of the systole (around 0.06 m/s) and decreased quickly to 0.02 m/s until the middle of the systole. The ES velocities were comparable in all three cases. The time courses of the velocities in all three local directions for Case 1 and Case 6 are visualized in Fig. 5, on the right. The visualization of Case 5 was omitted, since it was comparable to Case 6.

The longitudinal strain of the LA increased slower during the systolic period for Case 1 compared to Case 5 and Case 6. The maximal longitudinal strain of the LA occurred at ES and it was 20% for all three cases.

We did not observe any major differences in the measures between both hypertrophic geometries and therefore, in the following, we omit the less hypertrophic geometry (HCM 1) for further comparisons.

### Altered mechanics due to the stiffness of LV

We quantified isolated changes in the passive force by comparing the values of the deformation measures in the simulation with control stiffness (Case 1) to the one with increased stiffness (Case 2), both with the control geometry.

At ED, the regional wall thickening was comparable between Case 1 and Case 2. At ES, it differed, since the range of the thickening values was reduced: the maximum decreased more than the minimum did, when the stiffness of the tissue was increased (Case 1: 18.7%–50.0% and Case 2: 16.9%–35.8%). In particular, the wall thickening of the segments in the free wall of the LV (5, 6, 11 and 12) were diminished compared to the one in the septal segments (2, 3, 8, and 9). This led to more homogeneous distribution of the wall thickening among all segments when the stiffness of the tissue was increased (Fig. 2, Case 1 vs. Case 2).

**Fig. 7:**
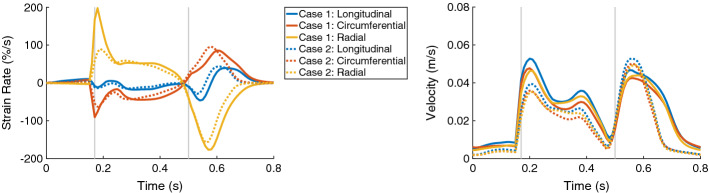
The time courses of the global strain rates (longitudinal, circumferential, and radial) are on the left and the global velocities (longitudinal, circumferential, and radial) are on the right, for Case 1 (solid lines) and Case 2 (dotted lines). In each plot, the first vertical line (at 0.17 s) indicates ED and the second line (at 0.5 s) ES. Case 1: control case; Case 2: increased stiffness

Similar to the wall thickening, the regional strain in all directions (longitudinal, circumferential, and radial) at ES had reduced extent of the values when the stiffness was increased. Therefore, a more homogeneous distribution of the strain among the all segments at ES was present for Case 2 compared to Case 1 (Fig. 6). The global radial strain rate at the beginning of the systolic period was halved when the stiffness was increased (Fig. 7, left).

During the entire systolic period, the global velocities in all directions reduced when the stiffness was increased. During the diastolic period, the maximal velocities in all local directions slightly increased as the stiffness increased (Fig. 7, right). Furthermore, the velocities decreased quicker as the stiffness was increased.

**Fig. 8:**
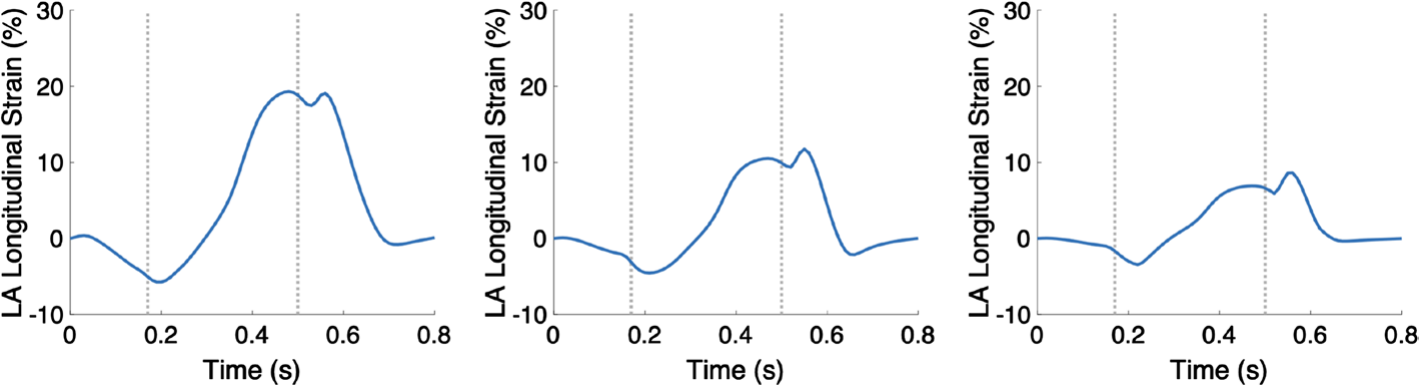
The time courses of the LA longitudinal strain for Case 1, 2, and 13 (left, middle, and right, respectively). In each plot, the first vertical line (at 0.17 s) indicates ED and the second line (at 0.5 s) ES. Case 1: control case; Case 2: increased stiffness; Case 13: virtual HCM case (all pathological changes included)

The longitudinal strain of the LA strongly decreased for the case of increased stiffness: 20% for Case 1 to 10% for Case 2 (Fig. 8, left and middle, respectively).

A comparison of the values of the deformation measures in the simulation with control stiffness (Case 6) and the one with increased stiffness (Case 7), both with the hypertrophic geometry (HCM 2), confirmed these results.

### Altered mechanics due to the active force development of LV

We quantified isolated changes in the maximal active force developed in the tissue by comparing the values of the deformation measures of the simulation with control active force (Case 6) to the one with decreased active force (Case 8), both with the hypertrophic geometry (HCM 2).

At ED, the regional wall thickening was comparable between Case 6 and Case 8. At ES, the regional wall thickening differed—the range of the thickening values remained similar, while the maximum decreased when the force was decreased. At the same time, the distribution of the thickening values among the AHA segments was retained when the active force was decreased (Fig. 2).

The regional circumferential and radial strain at ES had similar range of the values ($\approx$13.5%) for Case 6 and Case 8. The ranges at ES were shifted—the circumferential strain indicated less shorting of the tissue (values are higher, since they are negative) and the radial strain indicated less elongation of the tissue (values are lower, since they are positive) (Fig. 6).

**Fig. 9:**
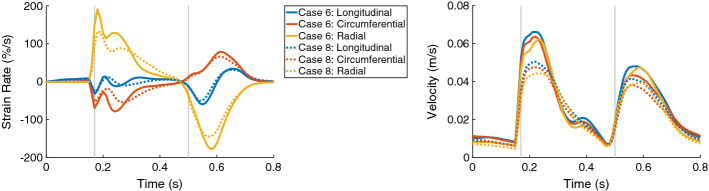
The time courses of the global strain rates (longitudinal, circumferential, and radial) are on the left and the global velocities (longitudinal, circumferential, and radial) are on the right, for Case 6 (solid lines) and Case 8 (dotted lines). In each plot, the first vertical line (at 0.17 s) indicates ED and the second line (at 0.5 s) ES. Case 6: hypertrophic geometry (17 mm); Case 8: hypertrophic geometry (17 mm), decreased active force

The maximal global circumferential and radial strain rates during the entire heart cycle differed between between Case 6 and Case 8. The circumferential strain rate indicated slower decrease of the strain, while the radial strain rate indicated slower increase of the strain when the active force was reduced (Fig. 9, on the left).

The maximal velocities in all directions (longitudinal, circumferential, and radial) were reduced during the entire heart cycle when the active force was reduced (Fig. 9, on the right).

The longitudinal strain of the LA was lower for the case of reduced active force—20% for Case 6 to 15% for Case 8.

A comparison of the values of the deformation measures in the simulation with control active force (Case 1) and the one with decreased active force (Case 3), both with the control geometry, confirmed these results.

### Altered mechanics due to fiber disarray of LV

We quantified isolated changes in the FO of the LV by comparing the values of the deformation measures in the simulation with control FO (Case 6) to the one with disarrayed FO (Case 10).

The RMSD of the global wall thickening was less than 3.2% for the entire heart cycle (Case 6 vs. Case 10: 1.1% (ED) and 3.2% (ES)). Similarly, the regional wall thickening showed minor differences. At ES, the range of the values for Case 6 was 18.1%–51.3%, and for Case 10, it was 19.7%–49.4%, while differences occurred mainly in the basal segments (1–6).

The regional longitudinal strain at ES was not influenced by the disarrayed FO. The regional circumferential and radial strain at ES had smaller extend of the values for the case with disarrayed FO. The difference was more pronounced for the circumferential strain—the range of the values for Case 6 was −15.5%–−3.4%, and for Case 10, it was −12.9%–−2.7%. Therefore, the circumferential strain indicated less shorting of the tissue (values are higher, since they are negative) and the radial strain indicated slightly less elongation of the tissue (values are lower, since they are positive) (Fig. 6).

The regional circumferential and radial strain rate indicated a slower change in the strains at the beginning of the systolic period. The values of the longitudinal strain rate were similar—the systolic RMSD was 2.2%.

The velocities in all three directions were comparable—the RMSD was 0.003 m/s during the systole and 0.001 m/s during the diastole.

The longitudinal strain of the LA slightly decreased when the FO was disarrayed—from 20% to around 18%.

A comparison of the values of the deformation measures in the simulation with control FO (Case 7) to the one with disarrayed FO (Case 11), both cases with increased stiffness, confirmed these results.

### Altered mechanics due to combination of pathological model components

We compared the deformation of the control case (Case 1) to the virtual HCM heart (Case 13), which was the combination of hypertrophic geometry, stiffened passive behavior, decreased active force development, and disarrayed FO.

The RMSD of the global wall thickening was 7.6% for the systole and 7.7% for the diastole. The maximum of the regional wall thickening decreased—from 50.0% for the control case to 27.7% for virtual HCM heart. As previously described, a decrease in the maximum was observed when the active force was decreased (e.g., Case 6 vs. Case 8). Furthermore, the extent of the wall thickening values reduced, since the maximum decreased more than the minimum (at ES, the wall thickening ranged from 18.7% to 50.0% for Case 1 and from 10.4% to 27.7% for Case 13). As previously described, a decrease in the range of the wall thickening, which led to a more homogeneous distribution of the wall thickening, was observed when the stiffness of the tissue was increased (e.g., Case 1 vs. Case 2).

**Fig. 10:**
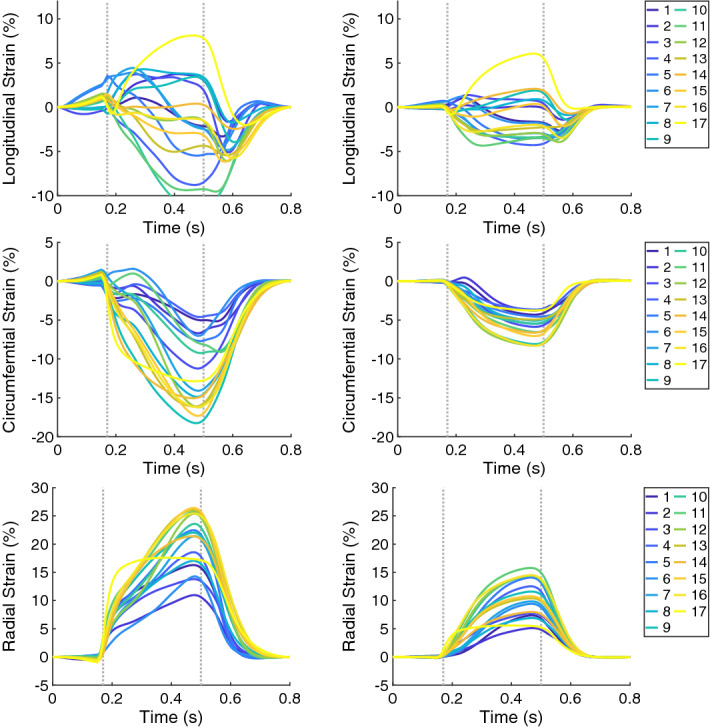
The time courses of the regional strains (in each of the 17 AHA segments) for Case 1 and Case 13 on the left and right, respectively. The longitudinal, circumferential, and radial strain are in shown in the first, second, and third rows, respectively. In each plot, the first vertical line (at 0.17 s) indicates ED and the second line (at 0.5 s) ES. Case 1: control case; Case 13: virtual HCM case (all pathological changes included)

Similar to the wall thickening, the regional strain values in all directions (longitudinal, circumferential, and radial) were closer to zero for the HCM case compared to control case (Fig. 10). The extent of the strain values reduced as well—the ES longitudinal strain was between −11.0% and 7.8% for Case 1 and between −3.5% and 5.5% for Case 13. At ES, the circumferential strain for Case 1 ranged from −17.8 to −4.5% and for Case 13: from −8.0% to −3.5%. The radial strain for Case 1 ranged from 10.6% to 25.7% and for Case 13: from 4.8 to 14.8% (Fig. 6).

**Fig. 11:**
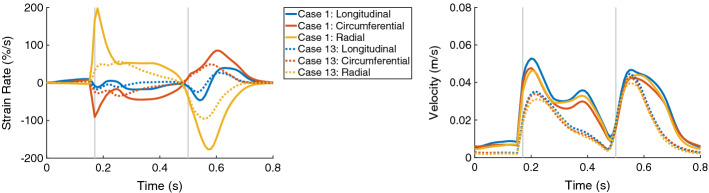
The time courses of the global strain rates (longitudinal, circumferential, and radial) are on the left and the global velocities (longitudinal, circumferential, and radial) are on the right, for Case 1 (solid lines) and Case 13 (dotted lines). In each plot, the first vertical line (at 0.17 s) indicates ED and the second line (at 0.5 s) ES. Case 1: control case; Case 13: virtual HCM case (all pathological changes included)

The global strain rates reduced for the HCM heart compared to control case for the entire heart cycle (Fig. 11, left). For each direction, the RMSD of the strain rates were similar for the diastolic and systolic period—around 12% in longitudinal direction, 23% in circumferential direction, and 41% in radial direction. During the systolic period, the major difference was observed during the first half of the systole. The global velocity in all three directions differed as well during the systolic and diastolic period—the RMSD was between 9.0% and 10.5% during the diastole and between 11.3 and 14.4% during the systole (Fig. 11, right).

The longitudinal strain of the LA strongly decreased—from 20% for Case 1 to 8% for Case 13 (Fig. 8, left and right, respectively).

### Overview of the results

Table 2 provides an overview of the results. Note that the measures which are negative indicate less or slower deformation as they increase—e.g., an increase in the diastolic radial strain rate indicates slower relaxation.

**Table 2:** Effect of model changes reflecting different pathological mechanisms (columns) on phenotypic mechanical markers (rows). WT = wall thickness, FA = fractional anisotropy, and Comb. = combination

Metrics	Time period	WT $\uparrow$	Passive force $\uparrow$	Active force $\downarrow$	FA $\uparrow$	Comb.
Wall thickening	Systolic (ES)	$\uparrow$ ($\approx$)	$\downarrow$ ($\downarrow$)	$\downarrow$ ($\downarrow$)	$\approx$ ($\approx$)	$\downarrow$ ($\downarrow$)
	Diastolic (ED)	$\approx$ ($\approx$)	$\approx$ ($\approx$)	$\approx$ ($\approx$)	$\approx$ ($\approx$)	$\approx$ ($\approx$)
Strain (L)	Systolic (ES)	$\uparrow$ ($\uparrow$)	$\uparrow$ ($\uparrow$)	$\approx$ ($\approx$)	$\approx$ ($\approx$)	$\downarrow$ ($\downarrow$)
	Diastolic (ED)	$\approx$ ($\approx$)	$\approx$ ($\approx$)	$\approx$ ($\approx$)	$\approx$ ($\approx$)	$\approx$ ($\approx$)
Strain (C)	Systolic (ES)	$\uparrow$ ($\approx$)	$\uparrow$ ($\uparrow$)	$\uparrow$ ($\uparrow$)	$\uparrow$ ($\approx$)	$\uparrow$ ($\uparrow$)
	Diastolic (ED)	$\approx$ ($\approx$)	$\approx$ ($\approx$)	$\approx$ ($\approx$)	$\approx$ ($\approx$)	$\approx$ ($\approx$)
Strain (R)	Systolic (ES)	$\uparrow$ ($\approx$)	$\downarrow$ ($\downarrow$)	$\downarrow$ ($\downarrow$)	$\downarrow$ ($\approx$)	$\downarrow$ ($\downarrow$)
	Diastolic (ED)	$\approx$ ($\approx$)	$\approx$ ($\approx$)	$\approx$ ($\approx$)	$\approx$ ($\approx$)	$\approx$ ($\approx$)
Strain rate (L)	Systolic (ES)	$\approx$ ($\approx$)	$\approx$ ($\approx$)	$\approx$ ($\approx$)	$\approx$ ($\approx$)	$\approx$ ($\approx$)
	Diastolic (ED)	$\approx$ ($\approx$)	$\approx$ ($\approx$)	$\approx$ ($\approx$)	$\approx$ ($\approx$)	$\downarrow$ ($\approx$)
Strain rate (C)	Systolic (ES)	$\downarrow$ ($\approx$)	$\uparrow$ ($\approx$)	$\uparrow$ ($\approx$)	$\approx$ ($\approx$)	$\uparrow$ ($\approx$)
	Diastolic (ED)	$\approx$ ($\approx$)	$\uparrow$ ($\uparrow$)	$\downarrow$ ($\uparrow$)	$\approx$ ($\approx$)	$\downarrow$ ($\downarrow$)
Strain rate (R)	Systolic (ES)	$\uparrow$ ($\approx$)	$\downarrow$ ($\approx$)	$\downarrow$ ($\approx$)	$\approx$ ($\approx$)	$\downarrow$ ($\approx$)
	Diastolic (ED)	$\approx$ ($\approx$)	$\uparrow$ ($\downarrow$)	$\uparrow$ ($\downarrow$)	$\approx$ ($\approx$)	$\uparrow$ ($\downarrow$)
Velocity (L,C,R)	Systolic (ES)	$\uparrow$ ($\approx$)	$\downarrow$ ($\approx$)	$\downarrow$ ($\downarrow$)	$\approx$ ($\approx$)	$\downarrow$ ($\approx$)
	Diastolic (ED)	$\approx$ ($\uparrow$)	$\uparrow$ ($\downarrow$)	$\downarrow$ ($\approx$)	$\approx$ ($\approx$)	$\downarrow$ ($\downarrow$)
LA strain (L)	ES	$\approx$	$\downarrow$	$\downarrow$	$\approx$	$\downarrow$

## Discussion

### Main findings

#### Altered mechanics due to the wall thickness of LV

During the systolic period, the time course of the wall thickening and strain is altered between the control geometry and the hypertrophic geometries in all AHA segments. At ES, the circumferential and radial strain had only minor differences between the control geometry and the hypertrophic geometries, while the longitudinal strain indicated less shortening as the WT increased. The myocardial velocities for both hypertrophic geometries are increased during the systolic period compared to the control case.

#### Altered mechanics due to the stiffness of LV

An increased stiffness of the tissue of the LV led to more homogeneous wall thickening among the AHA segments as well as equalized the strains at ES. The strain rates and velocities were reduced during the systole, especially visible at the beginning of the systole. In contrast, the maximal velocities during the diastole increased when the stiffness was increased and reduced quicker in the stiffer tissue. The longitudinal strain of the LA was halved when the stiffness of the tissue was increased.

#### Altered mechanics due to the active force development of LV

A reduced maximal active force development in the tissue of the LV results in reduced wall thickening as well as reduced circumferential and radial strain at ES in all AHA segments. It also results in reduced strain rates and velocities in the entire heart cycle. In total, less deformation is available in the entire LV.

#### Altered mechanics due to fiber disarray of LV

Disarrayed FO in the mid-wall of the LV led to less deformation in circumferential direction—the strain indicated less circumferential shortening of the tissue. Furthermore, it led to slightly less deformation in radial direction. The strain rates and the velocities were not considerably changed.

#### Altered mechanics due to combination of pathological model components

For the combined HCM heart, a decreased and more homogeneous wall thickening was observed at ES compared to the control case. Furthermore, the strain in all three directions indicated less deformation for HCM case compared to the control case. The strain rate revealed slower shortening and elongation of the tissue during the entire heart beat, but especially visible during the beginning of the systole. The longitudinal strain of the LA was more than halved for HCM case compared to control case. In total, the deformation and its rate of the LV were diminished for the virtual HCM heart compared to the control case.

### Altered mechanics in HCM patients reported in the literature

In HCM patients, global strains and strain rates are reported to be significantly lower  [1, 11, 22], while Ito et al. [9] measured preserved (or even increased) circumferential shortening. Furthermore, altered myocardial velocities are detected in HCM patients compared to controls—global and segmental diastolic velocities are decreased and systolic longitudinal velocities were reduced in HCM [10]. In the LA, a higher minimum volume and a lower peak atrial longitudinal strain were measured in HCM compared to controls [11].

### Causes of altered mechanics in HCM patients reported in the literature

Previous studies have examined potential origins of altered cardiac mechanics in HCM patients in clinical studies. To the best of our knowledge, no computational study was conducted on this topic. In a cohort of 59 HCM patients, Urbano-Moral et al. [2] demonstrated the relation of a reduction in longitudinal shortening of the LV and the extent of hypertrophy. Furthermore, the reduction of the global strain and strain rate was correlated with the mean WT [23]. In a clinical study, Villemain et al. [5] suggested that altered LV relaxation might result from increased myocardial stiffness. Hoskins et al. [6] hypothesized that reduced active force might contribute to systolic dysfunction in HCM patients.

In an HCM patients’ heart, distinct underlying phenomena are present simultaneously, but are differently pronounced. Thus, the effects of these phenomena cannot be clearly separated from measurements obtained in clinical studies. In contrast, in the presented numerical study, we could relate the observed alterations of mechanics to their underlying causes.

### Identification of underlying pathophysiology

An alteration of the wall thickening and the strain time course during the systole can be related to hypertrophic LV walls. Longitudinal strain, which indicates less shortening, is as well related to hypertrophic LV walls. Reduced ES wall thickening and strain values (circumferential and radial) in all segments can be related to less active force development in the LV. A homogeneous distribution of the ES wall thickening and strains among all AHA segments can be related to stiffer tissue. A reduction of the circumferential strain can be attributed to the fiber disarray in the mid-wall of the LV, but also to less active force development or increased stiffness of the tissue.

Strain rates that are reduced during the entire systole and strongly reduced during the beginning of the systole can be traced back to increased tissue stiffness. Strain rates that are reduced during the entire heart cycle are caused by reduced active tension developed in the tissue.

An increase in the myocardial velocities in all directions during the systolic period can be related to hypertrophic walls of the LV. In contrast, these velocities decreased during the systolic period when the stiffness of the tissue was increased. Additionally, an increase in the velocities during the diastolic period combined with a rapid decay of the velocities can be as well related to an increased stiffness of LV tissue. A reduction of the active tension development did not affect the velocities during the diastolic period.

A reduced longitudinal strain of the LA at ES was present in case of increased stiffness of the tissue or reduced active tension development.

### Comparison between simulated and clinically measured deformation

#### LV volume

**Fig. 12:**
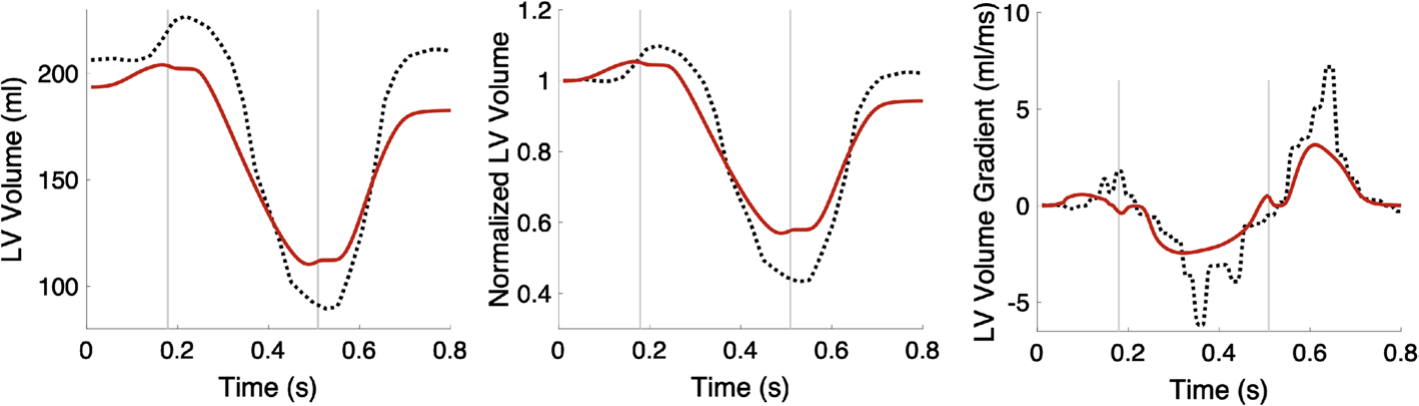
LV volume from the simulation of the control case (red curve) vs. LV volume extracted from Cine MRI data (dotted black line) Left: absolute volumes in ml. Middle: normalized volumes. Right: the gradient of the volume curves. In each plot, the first vertical line (at 0.17 s) indicates ED and the second line (at 0.5 s) ES

A comparison between the LV volume of the control case and the volume curve extracted from short-axis Cine MRI data is shown in Fig. 12. The absolute volume curves show that the initial volume of the virtual control heart is lower than the one measured in the clinical data, the ES volume is higher in the virtual control heart, and the atrial kick contributes less to the diastolic filling of the LV in the simulation compared to reality. However, the morphology of the normalized LV volumes and the gradient of the volume curves is comparable between simulated and MRI data. Still, the maximal and the minimal values of the volume curves and their gradients differ between the simulated and MRI data. We do not have any clinically measured strain or pressure values of the control heart, and therefore, a comparison against literature values was conducted.

#### Wall thickening

A segmentation of the endocardial and epicardial surfaces of the LV for the entire heart cycle is time-consuming and user dependent. Therefore, in clinical routine, the wall thickening is not commonly evaluated, but instead, the radial strain is used. On the virtual hearts, we applied two distinct methods to calculate the radial strain and the wall thickening. For both measures, we obtained comparable time course morphology and ES distribution for each case, but different maximal values [compare for Case 1 the wall thickening (max 50%) in Fig. 1, left and the radial strain (max 26%) in Fig. 10, left].

Kato et al. [24] showed that the radial thickening of the tissue arises due to the circumferential fiber shortening. This implies that fiber disarray in the mid-wall (fiber oriented in circumferential direction) will change the radial thickening and strain. In contrast, we observed a reduction of the deformation in circumferential direction, which was more pronounced than the reduction of the deformation in radial direction.

#### Strain and strain rate

The strain values obtained for the virtual control case (Case 1) indicated less shortening compared to healthy volunteers in all three directions [25]. Nevertheless, the regional values of the circumferential and radial strain of the virtual control heart are inside the ranges (mean±SD) provided in Table 4 in [25] but deviate from the mean values (e.g., the mean values of the regional circumferential strain were between −26 and −17% and the mean values of the regional radial strain were between 12% and 39%in [25]). Additionally, we obtained heterogeneous distributions among the AHA segments and heterogeneous values were as well measured—e.g., radial strain was 39 ± 21% in the anterior basal segment, and it was 12 ± 8% in the septum basal segment [25]. The heterogeneity in the circumferential strain was significant (p <0.05) [26].

The regional longitudinal strain in the virtual control heart strongly deviated from the ranges provided for the healthy volunteers (from −24 ± 11% to −13 ± 7%  [25]). For the virtual HCM heart, the longitudinal strain also deviated from literature values, which were around −11% for HCM hearts [11]. For each virtual heart, the values in the free wall were negative as well, but closer to zero (Fig. 6), while the positive values of the longitudinal strain were mainly in the septal segments. The torsion of the ventricle, which is pronounced in the apical region, leads to an elongation of the septal segments—the apex pulls the septal segments as it rotates. At the same time, the free wall shortened. The simultaneous occurrences of positive and negative values of the longitudinal strain in different segments cancel each other out when the global measures of strain and strain rate are calculated. Therefore, the value of the longitudinal strain rate was around 0 (Fig. 5).

For the virtual HCM heart, the strain values indicated less (and slower) shortening in the circumferential direction and less (and slower) thickening in the radial direction of the tissue compared to the virtual control case, in agreement with [1, 11, 22]. In contrast to Ito et al. [9], the circumferential strain of the virtual HCM heart indicated clearly reduced shortening.

The stiff heart syndrome (cardiac amyloidosis) can be identified by the longitudinal strain, which shows a strong gradient between the apex and base regions (apical sparing) [27, 28]. In the stiffened virtual hearts (e.g., Case 2 and Case 7), we observed an opposite effect when the stiffness was increased, and the strain became more homogeneous.

#### Velocity

Li et al. [10] measured reduced global and segmental diastolic radial and longitudinal peak velocities in patients with HCM vs. controls. In contrast, we did not observe a reduction of any peak velocity during the diastolic period in our virtual HCM heart compared to the control heart. Nevertheless, we observed a decrease in these velocities when the active force was reduced (Case 3) and an increase when the stiffness was increased (Case 2). Therefore, we showed that the effects of these two pathologies cancel out in our virtual HCM heart to obtain the same peak diastolic velocity as in the control case (Fig. 11, right). If we would either further reduce the active force in the LV or less deviate from the control stiffness (or both), we would obtain reduced diastolic velocities in line with [10].

When the stiffness of the tissue was increased, the amplitude of the diastolic velocity increased and also the morphology of the diastolic velocity course changed compared to the control case. Therefore, we confirm the suggestion of Villemain et al. [5] that modified LV relaxation might result from increased myocardial stiffness.

Furthermore, Li et al. [10] measured reduced peak velocities during the systole in patients with HCM in the longitudinal direction (radial peak velocities were comparable). We observed a reduction of the velocities during the systolic period as well but for all directions (longitudinal, circumferential, and radial). We did not compare the absolute values of velocities, since different imaging modalities provide different values for the velocities—feature tracking peak velocities are lower than directly measured tissue phase mapping velocities [10]. Hoskins et al. [6] hypothesized that reduced active force might contribute to systolic dysfunction in HCM patients. In agreement with [6], we observed reduced systolic function when the force was decreased—the velocities during the systole were decreased and the strain at ES was diminished.

In a cohort of healthy volunteers, similar global longitudinal and radial velocities during the systole were measured (longitudinal was 2.6 ± 0.55 cm/s and radial was 2.5 ± 0.36 cm/s) [25]. Likewise, we obtained similar global longitudinal and radial velocities. Nevertheless, the circumferential velocity was also in the same range, while Lin et al. [29] measured negative circumferential velocities, since the direction of deformation along the circumference was considered. We calculated the absolute values of these velocities which equal the speed of deformation in the circumferential direction. However, the direction of the deformation can be deduced from the strain values.

#### Left atrial strain

The LV of the virtual HCM heart deformed less compared to the control case, which led to reduced maximal longitudinal strain of the LA. Similar behavior was reported by Aly et al. [11]. Additionally, they discovered that LA dysfunction is present in HCM patients before global LV dysfunction can be measured. We could not reproduce this behavior in our model—in each case, in which LA longitudinal strain was reduced, the deformation of the LV was as well reduced. This reported statement demonstrates the importance of using a whole heart model, in which the deformation of all chambers is related.

#### Summary

The morphology of the clinically measured and the simulated LV volume curve was comparable. ED and ES volumes differed and the strain values indicated less shortening compared to measurements for healthy volunteers, but were inside the ranges reported in the literature. Furthermore, we obtained less longitudinal shorting of the LV compared to literature values. We showed that the effect of reduced active force and increase in the stiffness cancel each other out in our virtual HCM heart, thus yielding the same peak diastolic velocity as in the control case. We could confirm that modified LV relaxation results from increased tissue stiffness, as suggested in [5].

### Limitations

In the following, we describe limitations of our study and provide directions for potential improvements.

For the control geometry, the finite-element mesh of the ventricle was coarse—up to two elements in the transmural direction. The linear course of the FO is represented by two or three fibers in the transmural direction. Thus, we could not create a combination of control geometry and disarrayed FO. However, for the hypertrophic geometries, the spatial transmural discretization was sufficient to obtain a mid-wall disarrayed fiber orientation (Additional file 1: Figure S4).

Furthermore, the linearity of the solution to Laplace’s equation depends on the width of the domain between the boundaries [30]. Therefore, the course of the transmural coordinates used to define the LV mid-wall region close to the apex is not linear. In future, a linear course can be obtained by solving a trajectory distance equation in the LV [30].

The measure of FA was calculated on the fine geometry (also used to create the FO, Fig. 14) and delivered values close to one (0.95 ± 0.11) in the mid-wall ring of the control FO and 0.81 ± 0.25 for the disarrayed FO. Ariga et al. [4] measured for a control case an FA of 0.52 ± 0.03 and for HCM patients 0.49 ± 0.05, which are considerably lower values compared to the values in our virtual hearts. This is the result of a rule-based algorithm, since it creates idealized FO. Nevertheless, the difference between the mean FA of the control and the one of the disarrayed FO in the virtual heart is 0.14, which is much higher compared to this difference measured by Ariga et al. [4] (0.03). Therefore, we consider the cases with disarrayed FO to be representative for severely disarrayed FO.

For all numerical simulations, an identical input parameter set for the circulatory model was applied. It led to healthy systolic pressure (120 mmHg) for the virtual control heart, but to lower systolic pressures in the virtual HCM heart (70 mmHg). The pressure, applied on the endocardial surface, influences the deformation and, therefore, the evaluated measures. In both control and HCM cases, the ejection faction (EF) was lower compared to literature values—44% vs. 72% [31]. This indicates that the contraction force is lower in both atria and ventricles in the simulation. However, an increase of the active force will increase the EF but also the systolic pressure in the control case. A diminished deformation and reduced contraction results in a reduced EF and therefore in reduced strains, as discussed previously.

The active force model provided the force based on a predefined curve (force over time) and a maximum value of the force. A more complex model will adjust the intervals of the increase and the decrease of the force based on the velocities (e.g., Land et al. [32]) and, therefore, the duration of the contraction and relaxation of the LV. Ito et al. [9] reported that regional LV filling for HCM hearts was prolonged compared to control hearts and that the impairment of the diastolic relaxation is a major sign of HCM. We observed the major changes in the measures during the systole rather than diastole with exception of the strain rates—the RSMD are higher during the systole for the strains, velocities, and wall thickening. Additionally, we did not evaluate the diastolic relaxation time extent.

In general, the values of the metrics in the apex segment might be misleading, since the local longitudinal directions in the apex strongly deviate from the global longitudinal direction (Fig. 17). This is a result of the algorithm which creates the local directions. Instead, we could use the global longitudinal direction as a local one, but then the three local directions (longitudinal, circumferential, and radial) will not build an orthogonal system in each volume element and could not provide linearly independent information. We calculated of the strain based on the deformation tensor *F*. Werys et al. [33] used the Green–Lagrangian strain tensor $E = 0.5(F^TF-I)$ to derive the strain directly from the motion of the myocardium from cine MRI images. Santiago et al. [15] projected the components of the Green–Lagrangian strain tensor on a global longitudinal direction to obtain the strain. There is no agreement in the literature how to apply the deformation tensor to obtain the strain, and therefore, we would obtain different strain values depending on the definition of strain.

## Conclusions

We conducted an in-silico study on virtual human whole hearts to identify causes of altered mechanics in hypertrophic cardiomyopathy (HCM) hearts. We simulated the deformation resulting from combinations of physiological and pathological models and evaluated the mechanical behavior by local and global measures (wall thickening, strain, strain rate, and deformation velocities).

The presented study shows which pathological mechanisms are required to be present in the LV to obtain altered mechanics and how they affect the deformation measures. An increased wall thickness leads to deformation alteration during the systole, while the ES values are comparable to control case. Stiffer tissue equalizes the strains at ES, while reduced active force development reduces the deformation of LV. Disarrayed FO in the mid-wall did not influence the deformation of the LV. An inversion of these arguments allows to identify present pathological mechanisms in the tissue which cause an altered mechanical behavior.

In the clinical routine, it is cumbersome to directly measure underlying pathological mechanisms, and therefore, those, derived from a numerical simulation, might be a valuable information for clinicians and can contribute to a more accurate diagnosis in HCM patients.

## Methods

In the following, we describe the heart geometry and the numerical solver used to obtain the deformation of the entire heart for three heart beats. Then, we describe how we modeled fiber disarray as measured in HCM patients and introduce the physiological and pathological mechanisms included in the sensitivity analysis. Finally, we present the metrics which measure alteration of left ventricular mechanics.

### The geometrical model of the heart

**Fig. 13:**
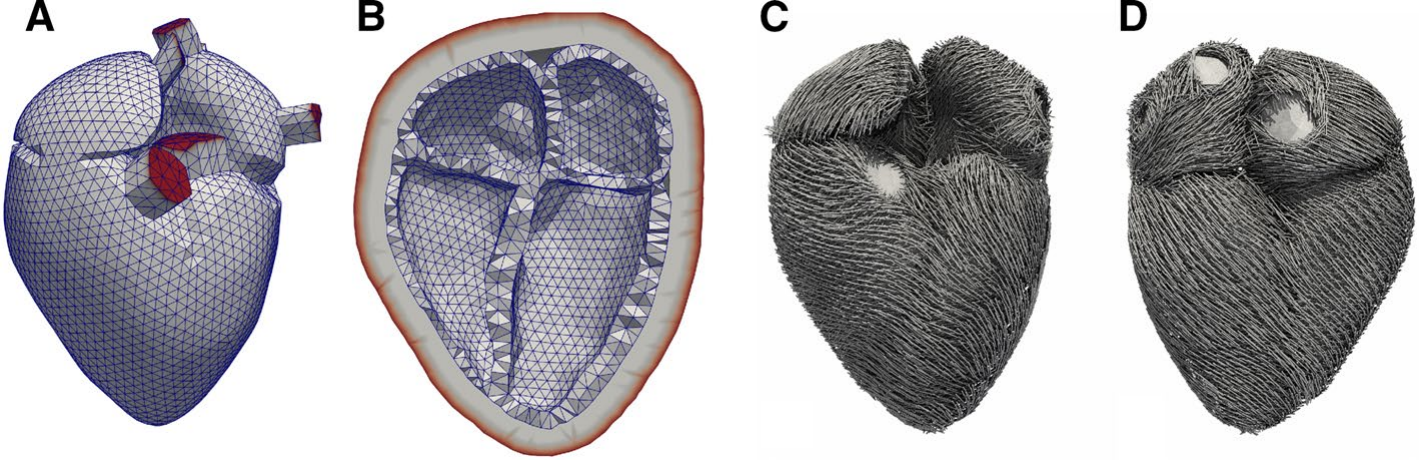
Geometrical model of the heart in mid-diastolic state. **A**  Anterior view of the four chambers and the visible trunks (aorta, pulmonary artery, superior pulmonary veins, and superior vena cava); **B** long axis cut of the four chambers and the pericardium with fixated nodes shown in red; **C** fiber orientation in anterior and **D** posterior view

The control geometry was based on MRI data of the whole heart, acquired from a healthy volunteer at University Hospital Heidelberg with a 1.5 T MR tomograph (Philips Medical Systems). Voxel spacing was 0.7 × 0.7 × 1.8 mm. The volunteer gave informed consent and the study was approved by the institutional review board. Images were segmented to obtain the endocardial and epicardial surfaces of the four chambers which provide the boundaries for the volume mesh of the myocardium. Additionally, the convex hull of the four chambers was calculated to serve as an inner surface of the pericardium. The volume between the myocardium and the pericardium was defined as fat tissue. The veins and arteries connected to the myocardium were represented in the model as trunks. The entire volume mesh consisted of 48,780 nodes and 90,801 cells. In the LV, there were two elements transmurally, which suffices to obtain correct deformation according to the convergence analysis conducted by Gerach et al. [34]. We used quadratic tetrahedral elements to discretize the volume and linear triangular elements for the surfaces. The nodes on the free ends of all trunks and the outside surface of the pericardial sac were fixated in all three directions to serve as a boundary condition for the model (Fig. 13A ,B).

### The numerical solver

To calculate cardiac deformation, we used the mechanical solver *CardioMechanics* [35], which was previously verified [36]. To describe the cardiac mechanics, the equation of balance of the linear momentum is solved by the Finite-Element Method. The governing equation ensures that all forces are in balance at all times during the heart beat. External forces arise outside the myocardium; internal forces arise inside the myocardium. To calculate the external forces, we included a closed-loop circulatory model and a pericardial model. The circulatory model provides a pressure–volume relation in the four chambers and delivers the pressure values, which are acting on the endocardial surfaces [34]. The closed-loop model ensures that the total blood volume in the circulatory system is preserved over several heart beats. The model is strongly coupled to the finite-element model as described by Gerach et al. [34]. The input parameters and the initial conditions are provided in Additional  file 1: Tables S1 and S2). The pericardial model represents the pericardial sac, in which the heart is embedded, and the surrounding tissue [35]. A sliding boundary condition is imposed between the inner surface of the pericardial model and the outer surface of the heart model. The pericardial model limits the motion of the heart by reducing the myocardial radial contraction and increasing the atrioventricular plane displacement. It delivers the forces acting on the epicardial surface of the entire heart. The internal forces are calculated by the combination of passive and active force models. The passive force model delivers the force arising from the intrinsic material properties of the myocardial tissue and is described by a constitutive relation. In this study, we applied the model of Guccione et al. [37] describing a hyperelastic, transversely isotropic material by the following strain energy function:

1 $$\begin{aligned} W&= \frac{C}{2} \left( e^Q - 1\right) + \frac{K}{2} \left( \text {det}(F) - 1\right) ^2, \nonumber \\ Q&= b_f E^2_{11} + b_t \left( E^2_{22} + E^2_{33} + E^2_{23} + E^2_{32}\right) + b_{ft} \left( E^2_{12} + E^2_{21} + E^2_{13} + E^2_{31}\right) , \end{aligned}$$

where *C*, $b_f$, $b_t$, and $b_{ft}$ are the parameters of the Guccione model, $E_{ij}$ $(i,j \in \left[ 1,2,3\right] )$ are elements of the Green strain tensor, $\text {det}(F)$ is the determinant of the deformation tensor, and *K* scales the incompressibility term. For the contractile tissue, $K = 10^6$ Pa was chosen and the parameters for the pericardium were chosen as in [35]. The fat tissue had the same passive properties as the pericardium. For the trunks, we applied the hyperelastic model of Mooney–Rivlin [38] with $C_1 = 14900$ Pa and $C_2 = 0$ Pa.

The active force model delivers the force acting along the fiber direction, which leads to fiber shortening and, therefore, to the contraction of the tissue. In this study, active force was described by a predefined curve as described by Stergiopulos et al. [39]. The normalized curve was scaled by a parameter, which determines the maximal active force. The ventricles were simultaneously activated 150 ms after the atria were activated (also simultaneously at 0 ms).

### Modeling of fiber disarray

The myocardial cells tend to align along their long axis to form bundles that are represented by fibers in the geometrical model. The FO determines the deformation of the tissue [40]. In our model, the FO in the atria was determined by the rule-based algorithm of Wachter et al. [41] (Fig. 13C, D). Fiber directions were assigned for each of the four quadrature points and for the centroid of each element.

Ariga et al. [4] visualized the myocardial microstructure of HCM hearts with DT-MRI. It allows quantifying the direction diffusion of water molecules by measuring the FA. A diffusion-weighted signal intensity is measured to construct the diffusion tensor (DT) [42]. The DT is a 3$\times$3 matrix obtained for each voxel and can be transformed to a diagonal matrix with its eigenvalues $\lambda _1$, $\lambda _2$, and $\lambda _3$ as diagonal elements [42]. The eigenvector belonging to $\lambda _1$ indicates the orientation of the long axis of the myocytes and $\lambda _1$, the magnitude of the diffusion in this direction. The other two eigenvectors are orthogonal to the primary one and define a transverse orthogonal plane. FA is calculated from the eigenvalues of the DT as follows [42]:

2 $$\begin{aligned} FA = \sqrt{\frac{3}{2}}\sqrt{ \frac{(\lambda _1 - D_{av})^2 + (\lambda _2 - D_{av})^2 + (\lambda _3 - D_{av})^2}{\lambda _1 ^2 + \lambda _2 ^2 + \lambda _3 ^2} }, \end{aligned}$$

where $D_{av}$ is the mean diffusivity; $D_{av} = (\lambda _1 + \lambda _2 + \lambda _3) / 3$. An FA value close to 0 corresponds to isotropic diffusion and therefore indicates tissue with variable FO. An FA value close to 1 corresponds to anisotropic diffusion and therefore indicates coherently aligned tissue [4].

Ariga et al. [4] measured reduced FA in the mid-wall ring (circumferentially aligned fibers) in the hearts of HCM patients compared to controls. We constructed a *virtual* DT to measure the FA in our computational heart model.

In the geometrical model, the FO is known for each element. Therefore, we estimated the diffusivity of the fibers $\lambda _1$ in finite-element regions to construct the virtual DT. We subdivided the LV in *N* regions ($v_i$, $i = 1, \dots , N$, *N* = 1500) of similar size (around $6 \times 20 \times 20$ mm) and calculated in each region the mean FO $f^{mean}_i$. For each element in the region ($e^k_i$, $k = 1, \dots , M$ with *M* the number of elements in the current region), we calculated the length of the projection of the fiber on the mean FO ($l^k_i$). Then, we set $\lambda _1$ of the region $v_i$ to the mean of these lengths across all elements in the region

3 $$\begin{aligned} \lambda _1 (v_i) = \frac{1}{M}\sum _{k=1} ^M l^k_i. \end{aligned}$$

The diffusivity in the other two directions was set to $\lambda _{2,3} (v_i) = 0.5( 1 - \lambda _1 (v_i))$. Finally, the values obtained for $\lambda _1$, $\lambda _2$, and $\lambda _3$ were used in Eq. 2 to obtain FA for the provided fiber configuration. Here again, for a coherent fiber arrangement in a specific region, we obtain $\lambda _1 = 1$, $\lambda _{2,3} = 0$ and therefore FA $= 1$. In a region of strongly disarrayed FO, we obtain $\lambda _{1,2,3} = 1/3$, and therefore, FA $=0$.

**Fig. 14:**
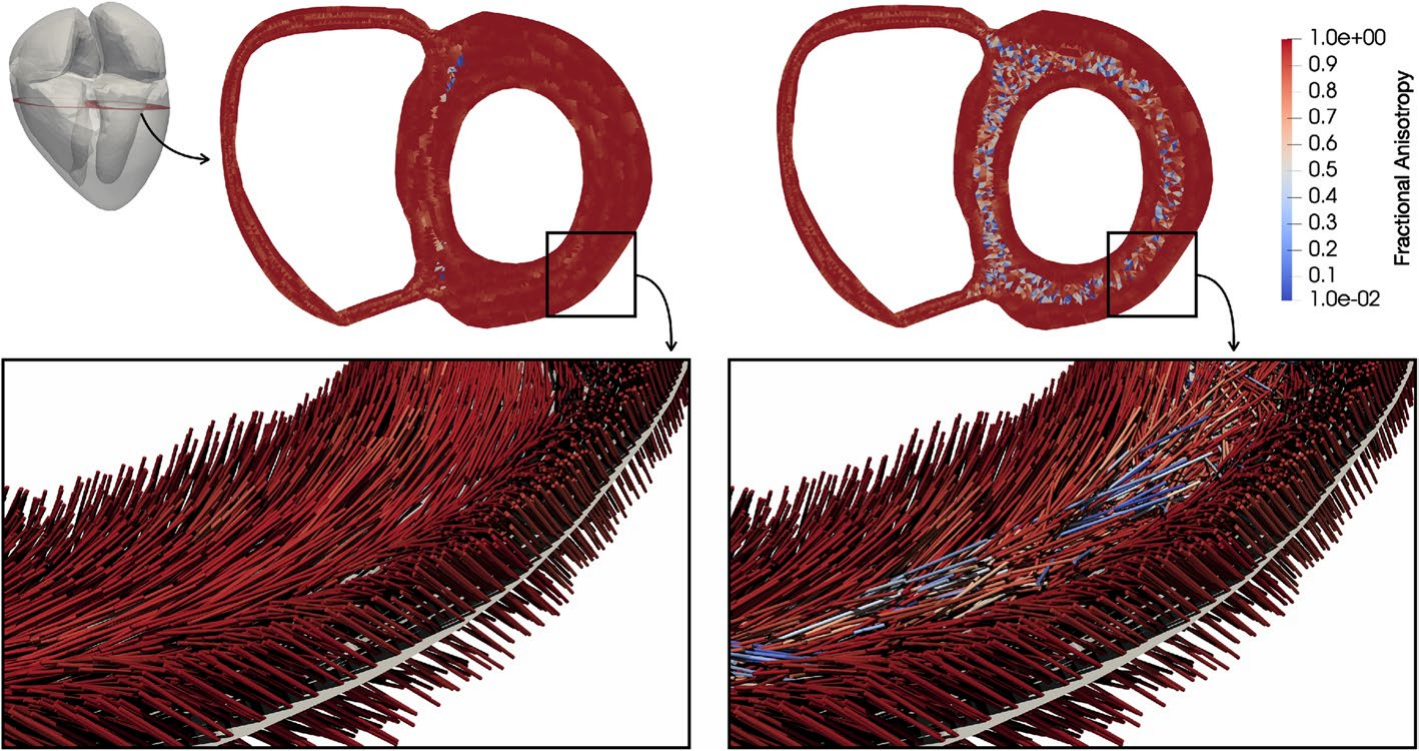
Fractional anisotropy and fiber orientation in a slice of the LV wall. FA is color-coded in short-axis slices. Bottom: close-up of fiber orientation in a part of the LV-free wall. Left: control fiber orientation; right: fiber disarray

**Fig. 15:**
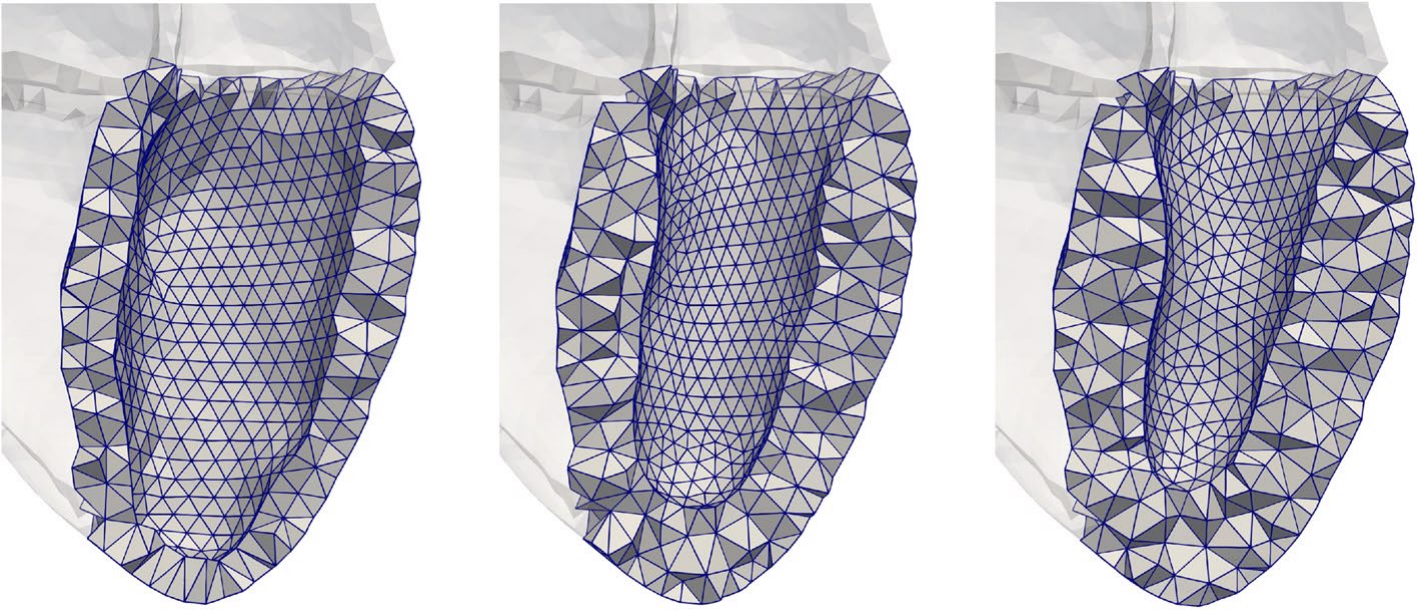
Three LV geometries clipped through their long axis. Left: initial geometry (mean wall thickness 10 mm). Middle and right: hypertrophic geometries (15 mm and 17 mm, respectively)

We adapted the fiber assignment algorithm to generate disarrayed FO in the mid-wall ring of the LV (Fig. 14). The mid-wall ring was defined to enclose all elements with transmural coordinates between 0.34 and 0.66. The transmural coordinate ranged from 1 on the endocardial surface to 0 on the epicardial surface, and was obtained by solving the Laplace’s equation in the volume. In this ring, the gradient value in each element was multiplied by a random number from a uniform distribution on the interval [0, 1] to obtain the distorted FO in this element. The sheet and sheet-normal directions were calculated to yield an orthonormal system together with the distorted fiber. Outside the mid-wall ring, the FO was assigned as in the control case. The FO were generated on a fine mesh (around 1 million elements). A nearest-neighbor interpolation transferred the FO to the coarse geometry used for the simulations (Fig. 15, right).

### Sensitivity analysis

We created geometries with increased WT of the LV, varied internal forces (passive and active), and distorted FO in the LV. For all cases, the external forces were defined using the same parameterization of circulatory and pericardial models.

#### Wall thickness

The WT (mean ± std) of the LV of the initial geometrical model (described in "The geometrical model of the heart"), was $10\,\pm \,2.3$ mm and its cavity volume was 193 ml. We added tissue on the endocardial surface to increase the thickness of the LV to 1) $15\,\pm \,3.3$ mm and 2) $17\,\pm \,4.1$ mm with a concomitant decrease of the LV volume (118 ml and 94 ml, respectively). In both cases, the added tissue was distributed concentrically. Figure 15 shows the three geometries with distinct WT. WT of the right ventricle and both atria were not modified compared to the initial geometrical model.

#### Passive forces

We varied the input parameters of the passive force model (described in "The numerical solver") which determines the tissue stiffness. To identify the parameters of the passive force model for the control case, we used a method based on the pressure–volume relation of LV as described previously [43] and obtained the following parameters for the Guccione model: $C =$ 309 Pa, $b_f =$ 17.8, $b_t =$ 7.1, and $b_{ft} =$ 12.4. In HCM myocardium, Villemain et al. [5] measured a fivefold increase of the stiffness compared to controls. Thus, we increased the parameter *C* which determines the global stiffness to 1545 Pa for the entire myocardium of all four chambers to capture increased tissue stiffness.

#### Active forces

We varied the input parameters of the active force model (described in *"*The numerical solver*"*). For the control case, the scaling parameter of the active force, $T^{\text {V}}_{\text {max}}$, was set to 100 kPa in both ventricles and $T^{\text {A}}_{\text {max}}$ = 35 kPa in both atria. These values were chosen to obtain a control systolic LV pressure of 120 mmHg in the control geometry. Hoskins et al. [6] measured a 40% decrease of the active force in HCM donor cells compared to controls. Therefore, we reduced the maximal active force to $T^{\text {V}}_{\text {max}} = 60$ kPa in both ventricles and $T^{\text {A}}_{\text {max}} = 21$ kPa in both atria.

#### Fiber orientation

We defined two configurations of FO in the LV: one control case and one representing fiber disarray. The control FO was determined by a rule-based algorithm based on Bayer et al. [44] with angles changing transmurally from 60$^\circ$ on the endocardium to $-60^\circ$ on the epicardium [45]. The algorithm (Bayer et al. [44]) was adapted to eliminate a discontinuity of fibers in the free walls and to yield a fiber rotation that is approximately linear across the wall.^1^

Ariga et al. [4] measured fiber disarray in HCM hearts. We modified the rule-based algorithm to yield disarrayed FO in the mid-wall ring of the LV (described in “Modeling of fiber disarray”). On the epicardium and endocardium, the same angles were used as in the control case. We quantified the disarray by calculating the FA in the mid-wall ring, which was 0.95 ± 0.11 for control and 0.81 ± 0.25 for the disarrayed FO (Fig. 14). The minimum FA (0.3) for the control fiber was observed at the junction of LV and RV.

### Evaluation metrics

We introduced metrics to evaluate the deformation of the LV and one metric for the left atrium (LA) based on common imaging-derived features [46, 47].

**Fig. 16:**
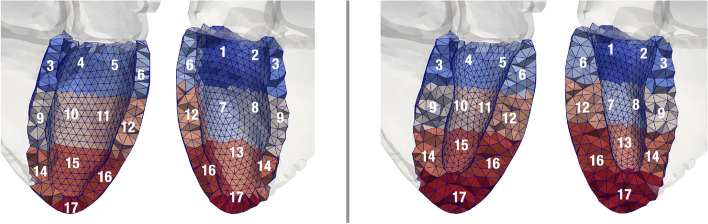
The division of the LV in 17 AHA segments. Each segment is numbered in both the anterior and posterior views. On the left of the gray separation line is the control geometry and on the right, the hypertrophic geometry (HCM 2). The apex segment (17) includes the endocardial apex

The following measures were evaluated *globally* (one value for the entire ventricle per time point) and *regionally* (one value per one of the 17 AHA segments [48] per time point, Fig. 16): *strain*, *strain rate*, *velocity*, and *wall thickening*.

**Fig. 17:**
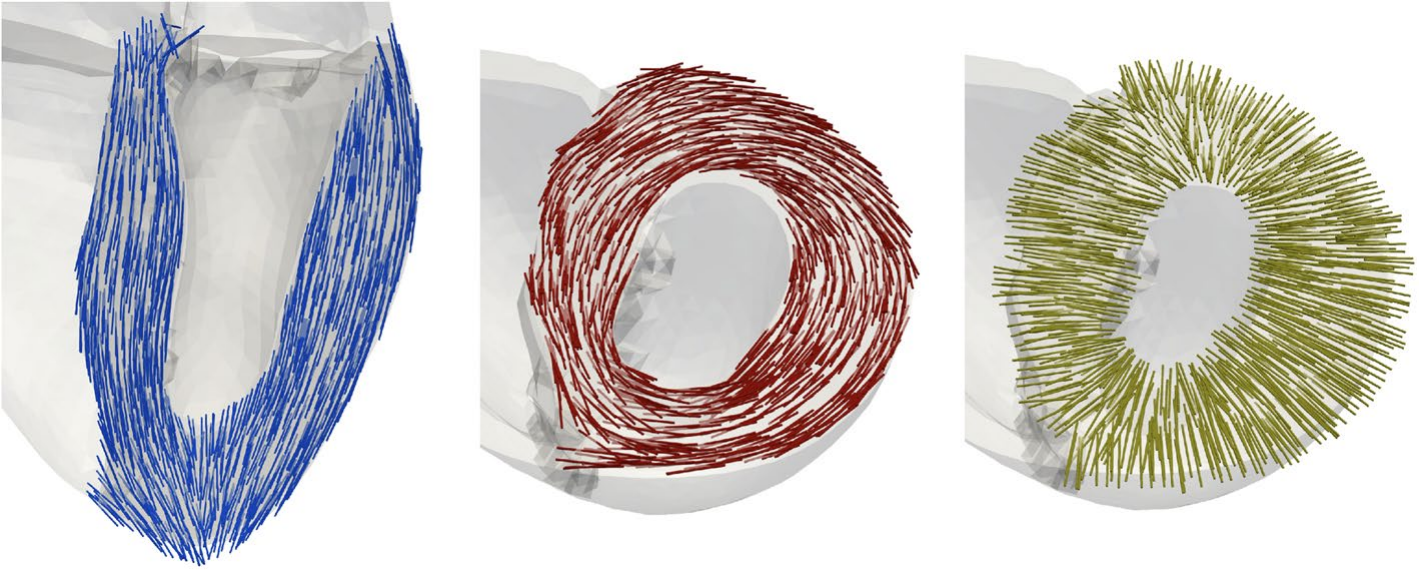
Local coordinate system spanned by longitudinal (blue, left), circumferential (red, middle), and radial (yellow, right) directions

The strain, strain rate, and velocities were calculated in a local heart coordinate system (R-Lo-C), spanned by radial, longitudinal, and circumferential directions [46] (Fig. 17). For every finite element in each geometry, these axes were calculated at the initial time point of the simulation and preserved over the heart beat. Regional and global measures were derived as the mean over all elements in the respective regions.

All measures were calculated during the systolic and during the diastolic period. The regional measures at end-systole and end-diastole are visualized in bull’s-eye displays [48]. Time of end-systole and end-diastole was determined based on the pressure–volume relation. For each global measure, we calculated the RMSD (root-mean-squared deviation) between each pair-wise combination of cases (Table 1) during the systolic and diastolic period.

#### Strain

Strain $\varepsilon$ (%) describes the change in length relative to the initial length (in one dimension). Positive strain values correspond to lengthening and negative to shortening [46].

In the numerical simulation, the deformation of the heart in each element of the mesh is characterized by a deformation gradient tensor *F* calculated for each time step of the heart beat. The FO $f_{d}$ in the deformed element is $F f_{init}$, where $f_{init}$ is the initial FO. The stretch of the deformed fiber is $\lambda (f_{d}) = \sqrt{f^T_{init} F^T F f_{init}}$ and the strain is $\varepsilon (f_{d}) = \lambda (f_{d}) -1$. The strain in the deformed sheet $\varepsilon (s_{d})$ and sheet-normal $\varepsilon (sn_{d})$ directions is calculated likewise.

The strain in the R-Lo-C system is then obtain by a coordinate transformation with matrix *T*. The matrix *T* transforms a vector with Cartesian coordinates (with respect to the standard basis of $\mathbb {R}^3$) into the local R-Lo-C coordinates. The rows of *T* are the radial, longitudinal, and circumferential vectors in the current element. By multiplication with the matrix *T* from the left, the normalized vectors pointing in the fiber ($f_{d}$), sheet ($s_{d}$) and sheet-normal ($sn_{d}$) directions in a deformed element are projected on each local direction vector, pointing in longitudinal, circumferential, and radial directions. Then, the projections are scaled by the strain obtained in the deformed element $\lambda (f_{d})$, $\lambda (s_{d})$, and $\lambda (sn_{d})$. The following equation provides the strain in the R-Lo-C system:

4 $$\begin{aligned} \varepsilon _{RLoC} = \left| T(f_{d}/\Vert f_{d}\Vert _2) \right| \varepsilon (f_{d}) + \left| T(s_{d}/\Vert s_{d}\Vert _2) \right| \varepsilon (s_{d}) + \left| T(sn_{d}/\Vert sn_{d}\Vert _2) \right| \varepsilon (sn_{d}). \end{aligned}$$

The strain in radial direction is the first entry of the transformed strain: $\varepsilon _{RLoC}(1)$, the strain in longitudinal direction is $\varepsilon _{RLoC}(2)$, and the strain in circumferential direction is $\varepsilon _{RLoC}(3)$.

#### Strain rate

Strain rate (%/s) is the speed at which the strain changes [46]. We calculated the strain rate $\dot{\varepsilon }_t$ at time *t* with $\Delta t =$ 0.01 s

5 $$\begin{aligned} \dot{\varepsilon }_t = (\varepsilon _t - \varepsilon _{t - \Delta t}) / \Delta t, \end{aligned}$$

where $\varepsilon _t$ is the strain at time *t*.

#### Velocity

Velocity (m/s) is the temporal change of the displacement. By solving the governing equation of the heart mechanics, we obtain the displacement and, thus, the velocity for every node and each time step [49].

To obtain the velocity of each finite element, the mean of the velocity over its four nodes was calculated for each of the three directions in the Cartesian coordinate system: $v = (v_x, v_y, v_z)$. To convert the Cartesian velocity into the local R-Lo-C system, the coordinate transformation with the matrix *T* was conducted analogous to the transformation of the strain: $v_{RLoC} = Tv^T$. Then, for the observed finite element at the current time point, the absolute value of the velocity in radial direction is $\left| v_{RLoC}(1)\right|$, in longitudinal direction $\left| v_{RLoC}(3)\right|$, and in circumferential direction $\left| v_{RLoC}(3)\right|$. In the following, the absolute value of the velocity will be referred to as *velocity*.

#### Wall thickening

The wall thickness (WT, $\omega$ in mm) of the LV was calculated as proposed by Yezzi et al. [50]. WT was obtained for every time step of the heart beat and each surface node. The wall thickening (in percent) for time *t* is then: $(\omega ^t - \omega ^{0})/\omega ^{0}$, where the upper index corresponds to the time with 0 denoting the initial WT.

#### Mechanics of left atrium

We defined a main longitudinal axis of the LA by calculating the mean of the local longitudinal directions over all elements in the LV. For every time point *t*, all nodes of the LA were orthogonally projected on the main axis. With the maximal Euclidean distance between any two projected points $l^t_{LA}$, the longitudinal strain for time *t* is then: $(l^t_{LA} - l^0_{LA}) / l^0_{LA}$, where the upper index 0 corresponds to the initial geometry configuration at time 0.

## Supplementary Information

**Additional file 1.** Additional tables and figures.

## Data Availability

The datasets used and/or analyzed during the current study are available from the corresponding author on reasonable request.

## Footnotes

1 Code available at https://github.com/KIT-IBT/LDRB_Fibers.
