# Supplementary material for: Causes of altered ventricular mechanics in hypertrophic cardiomyopathy: an in-silico study
Source: Biomed Eng Online. 2021 Jul 22;20:69. doi: 10.1186/s12938-021-00900-9 (PMC8296558; doi:10.1186/s12938-021-00900-9)
Supplement: Supplementary file 1 — Additional file 1. Additional tables and figures. [file 12938_2021_900_MOESM1_ESM.pdf]

Additional file 1: Causes of Altered Ventricular Mechanics in Hypertrophic Cardiomyopathy: an In-Silico Study

Autors: Ekaterina Kovacheva, Tobias Gerach, Steffen Schuler, Marco Ochs, Olaf Dössel, Axel Loewe

Circulatory system parameters and initial conditions Since in Gerach et al. [1], the parameters used in the numerical simulation are already provided in Table 4, here, we only highlight the parameters, which were different compared to these parameters:

Table 1 Circulatory system parameters, which were chosen differently compared to Table 4 in Gerach et al. [1]

Parameter	Value	Unit
R_{SysArt}	0.07	$\text{mmHg} \cdot \text{s} \cdot \text{ml}^{-1}$
C_{SysArt}	2.0	$\text{ml} \cdot \text{mmHg}^{-1}$
R_{SysPer}	0.9	$\text{mmHg} \cdot \text{s} \cdot \text{ml}^{-1}$
C_{SysVen}	100.0	$\text{ml} \cdot \text{mmHg}^{-1}$

Additionally, we provide the initial conditions for the circulatory system model:

Table 2 Initial conditions for the circulatory system model.

Parameter	Value	Unit
V_{tot}	5500	ml
V_{SysArt}	969	ml
V_{PulArt}	261	ml
V_{PulVen}	281	ml
p_{LV}	8.0	mmHg
p_{LA}	8.0	mmHg
p_{RV}	4.0	mmHg
p_{RA}	4.0	mmHg

RMSD matrices Figure 1 and Figure 2 provide RMSD matrices for pair-wise comparison of cases (row vs. column, Table 1 in the main document) for each evaluation metric. In each matrix, the systolic difference is above the diagonal and the diastolic difference is below the diagonal—e.g. the RMSD of the longitudinal strain rate (top right in Figure 1) between Case 1 and Case 13 during the systole is the last entry in the first row (11.7) and the RMSD between these cases during the diastole is the last entry in the first column (12.4) of the corresponding matrix.

Strains Figure 3 shows the bull's-eye displays of the longitudinal, circumferential and radial strain at ES for Case 3, 4, 5, 7, 9, 11 and 12.

Fiber orientation In Figure 4, we show a short axis clip through both ventricles of the coarse hypertrophic geometry HCM 2 used for simulations. On the left, the control fiber orientation are shown and on the right, the mid-wall fiber disarray.

Author details

References

1. Gerach, T., Schuler, S., Fröhlich, J., Lindner, L., Kovacheva, E., Moss, R., Wülfers, E.M., Seemann, G., Wieners, C., Loewe, A.: Electro-mechanical whole-heart digital twins: A fully coupled multi-physics approach. Mathematics **9**(11) (2021). doi:10.3390/math9111247

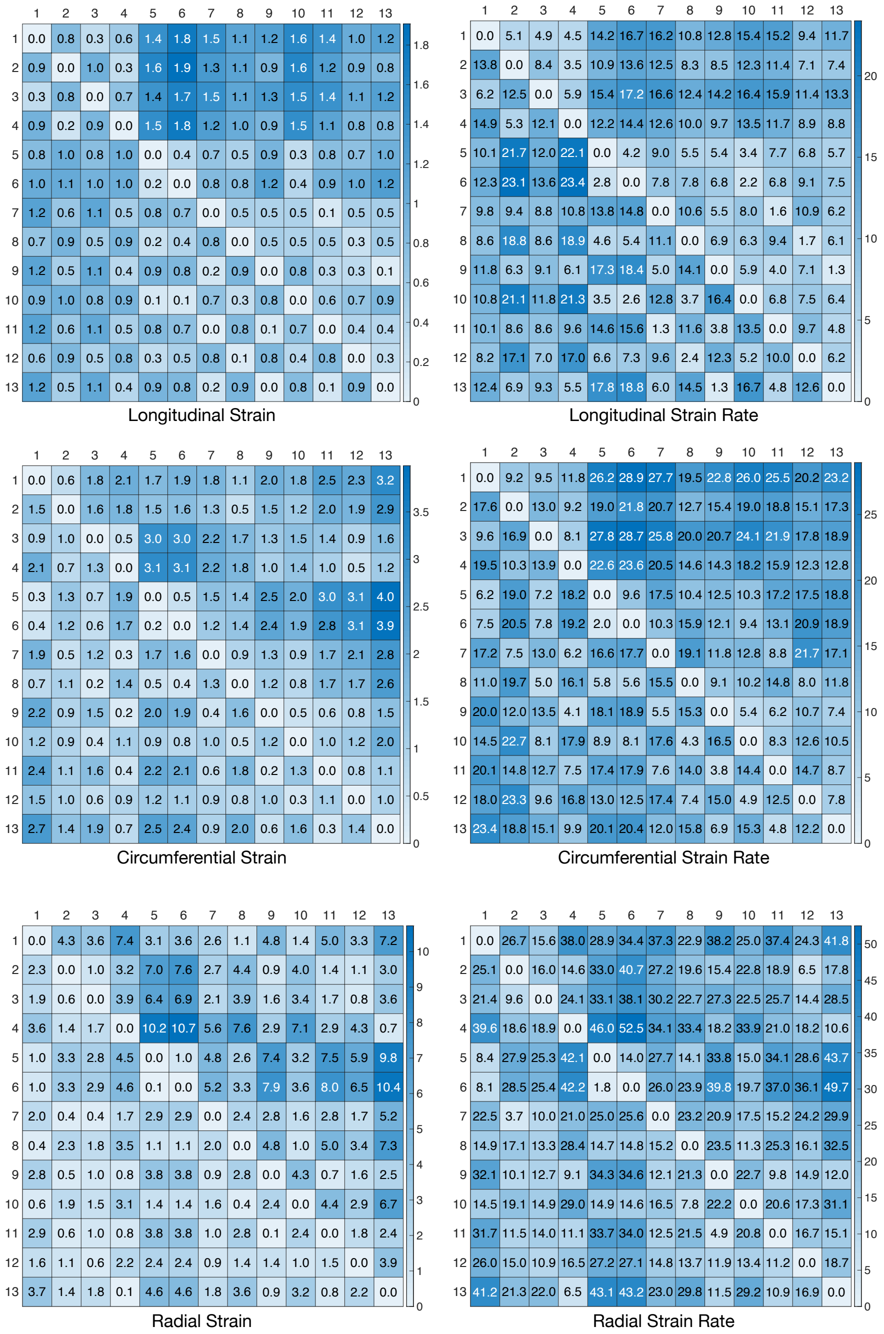

Figure 1 RMSD matrices for the strains(%) and strain rates (%/s) in the local directions.

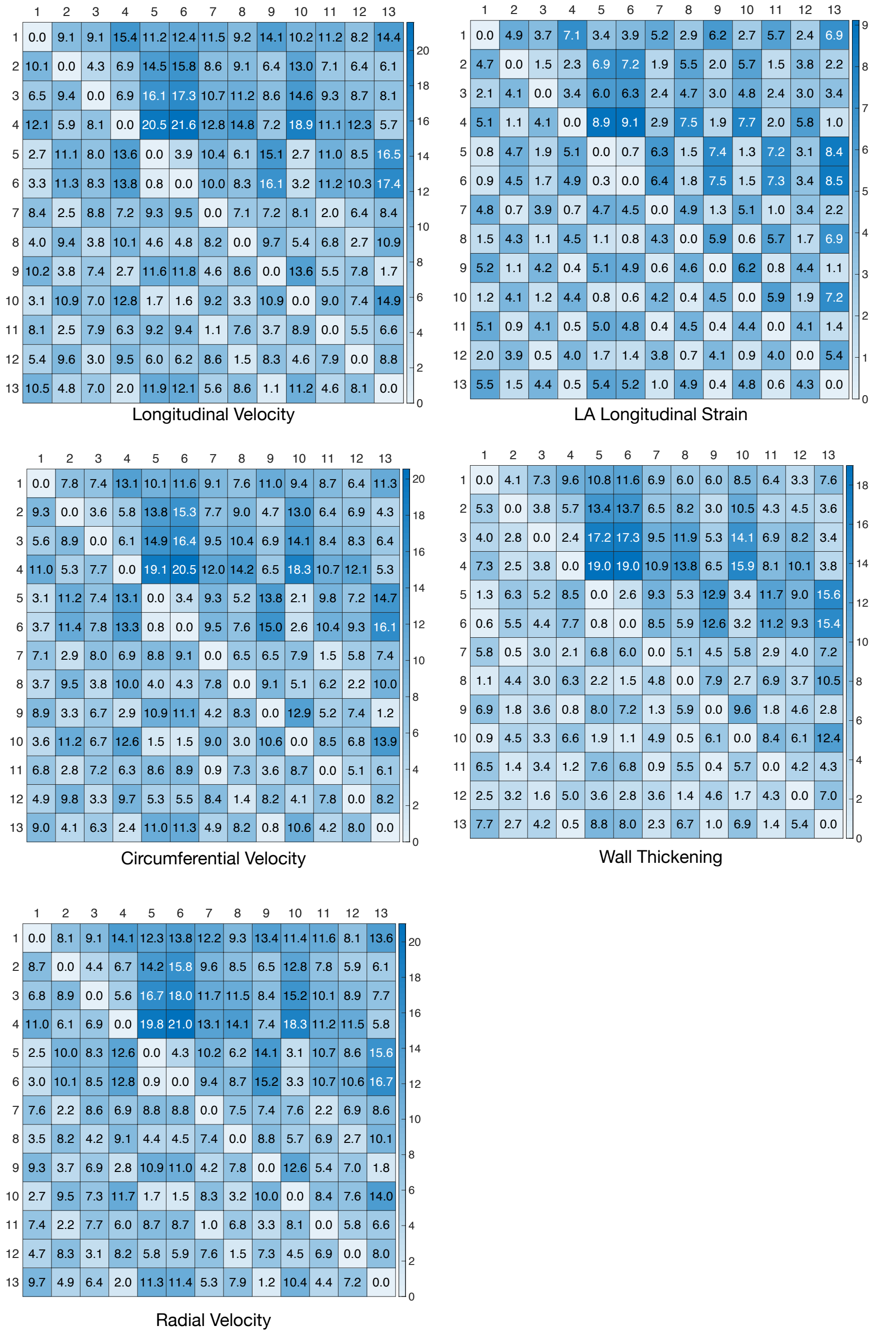

Figure 2 RMSD matrices for the velocities (mm/s) in the local directions, LA strain (%) and wall thickening (%).

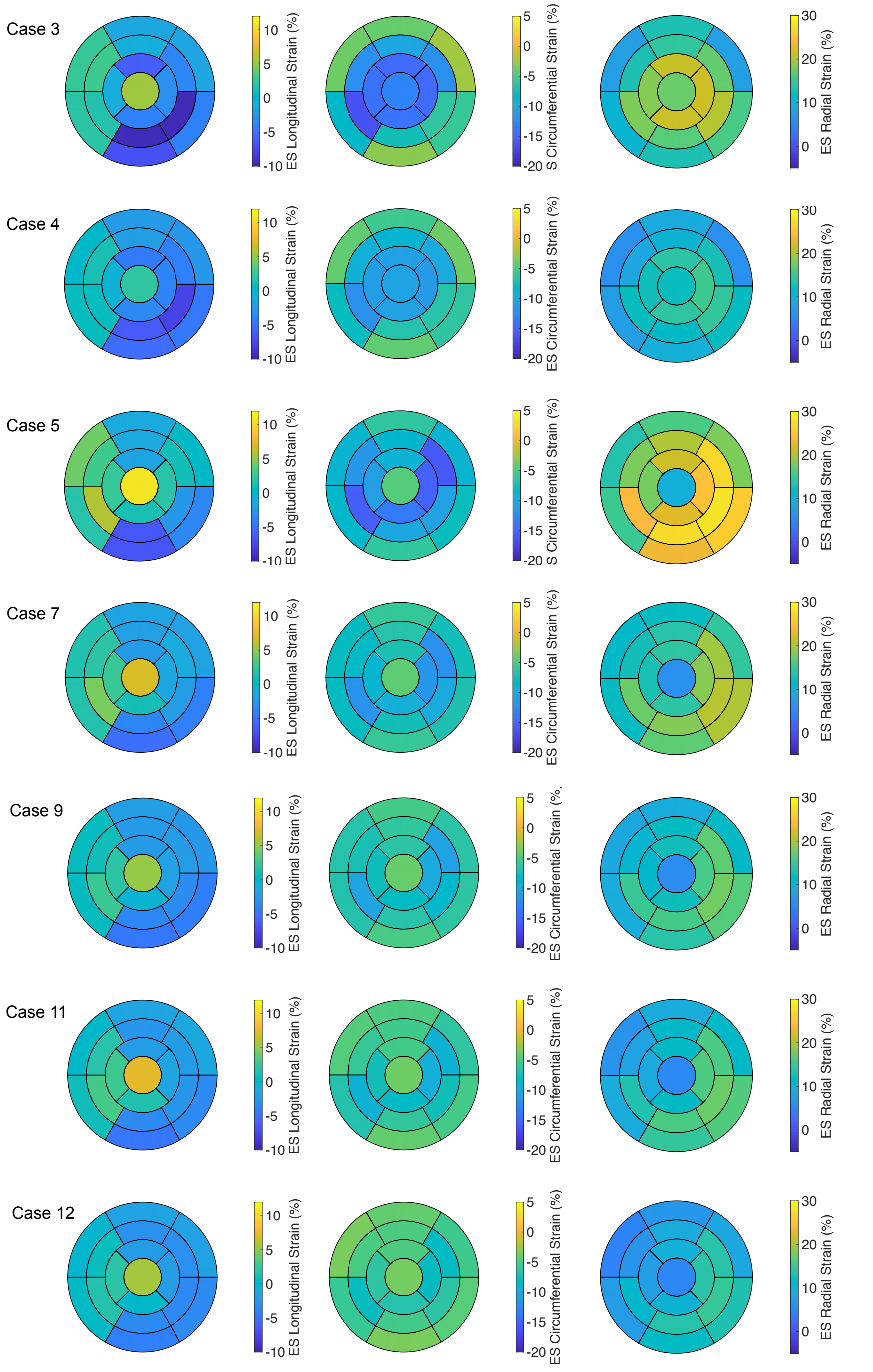

Figure 3 Bull's-eye displays for Case 3, 4, 5, 7, 9, 11 and 12 showing the longitudinal, circumferential and radial strain at ES (first, second and third column, respectively). Each row corresponds to one case. Case 3: decreased active force; Case 4: control geometry, increased stiffness and decreased active force; Case 5: hypertrophic geometry (15 mm); Case 7: hypertrophic geometry, increased stiffness; Case 9: hypertrophic geometry (17 mm), increased stiffness and decreased active force; Case 11: hypertrophic geometry (17 mm), fiber disarray; Case 12: hypertrophic geometry (17 mm), decreased active force, fiber disarray.

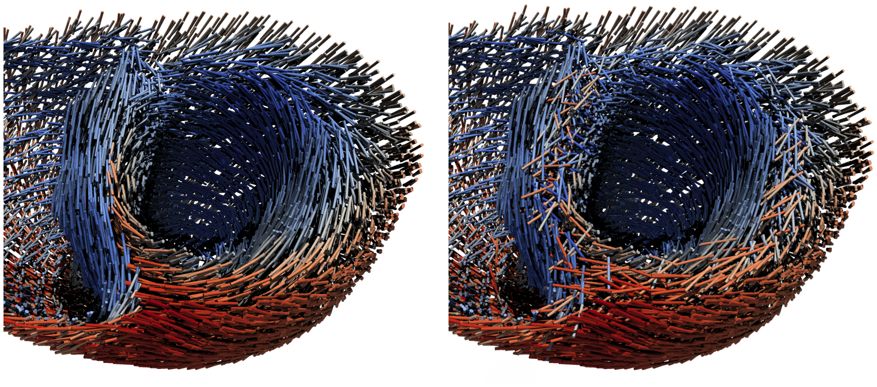

Figure 4 A short axis clip thorough both ventricles of the hypertrophic geometries HCM 2 with control fiber orientation (left) and with mid-wall fiber disarray (right). The color corresponds to the value of the x-coordinate of the orientation of the fibers.
